# Constructing LDPC Codes with Any Desired Girth

**DOI:** 10.3390/s21062012

**Published:** 2021-03-12

**Authors:** Chaohui Gao, Sen Liu, Dong Jiang, Lijun Chen

**Affiliations:** 1 State Key Laboratory for Novel Software Technology, Nanjing University, Nanjing 210046, China; dz1833008@smail.nju.edu.cn (C.G.); mf1833040@smail.nju.edu.cn (S.L.); 2 School of Internet, Anhui University, Hefei 230039, China

**Keywords:** error correction code, LDPC code, decoding

## Abstract

In wireless sensor networks, the reliability of communication can be greatly improved by applying low-density parity-check (LDPC) codes. Algorithms based on progressive-edge-growth (PEG) pattern and quasi-cyclic (QC) pattern are the mainstream approaches to constructing LDPC codes with good performance. However, these algorithms are not guaranteed to remove all short cycles to achieve the desired girth, and their excellent inputs are difficult to obtain. Herein, we propose an algorithm, which must be able to construct LDPC codes with the girth desired. In addition, the optimal input to the proposed algorithm is easy to find. Theoretical and experimental evidence of this study shows that the LDPC codes we construct have better decoding performance and less power consumption than the PEG-based and QC-based codes.

## 1. Introduction

The wireless sensor network (WSN) [1,2] is a distributed network, which consists of lots of small sensor nodes. Information from the environment is collected by the sensors and is delivered over the wireless channel to a central station where the desired data can be obtained by users. Due to the features such as scalability and self-organization, WSNs have a wide range of applications in the areas of medical care [3], target tracking [4], military [5], environmental monitoring [6] and so on [7,8,9]. Ensuring reliable communication is the most basic requirement of WSNs. However, since sensor nodes of WSNs are tiny in size and rely on lightweight batteries, they are heavily constrained by limited memory and processing power. In this case, using efficient block coding is needed. Low-density parity-check (LDPC) codes [10] have remarkable error-correcting performance, which can greatly improve the reliability of communication [11,12]. In addition, the low coding and decoding complexity of LDPC codes can reduce power consumption, thus extending the lifetimes of WSNs [13,14,15].

LDPC code was proposed by Gallager in 1962 [10]. It attracts an enormous amount of interest because of its capacity-approaching performance and low-complexity iterative decoding combined with the belief-propagation (BP) algorithm [16,17]. BP can provide optimum decoding when the LDPC code is cycle-free, so it is important to reduce the impact of cycles.

The girth is the low bound of the lengths of all cycles in an LDPC code, and a larger girth indicates that the code evades more short cycles. Algorithms based on progressive-edge-growth (PEG) pattern [18,19,20] and quasi-cyclic (QC) pattern [21,22] are the two main kinds of LDPC code construction algorithms used to create codes with large girth. In PEG-based algorithms, edges are greedily added into the LDPC code to maximize the length of the current shortest cycle such that the algorithms manage to make the girth larger. They are flexible and convenient to generate regular and irregular LDPC codes with short or medium code length. As for the QC-based algorithms, an LDPC code is divided into several parts and each part can be represented as either a zero matrix or a circulant permutation matrix. Eliminating short cycles to maximize the girth can be realized by changing the shift value [21] of every circulant permutation matrix. These algorithms are easy for hardware parallel implementation and the codes they constructed are space-saving. Despite these strengths, neither PEG-based nor QC-based algorithms can overcome some shortcomings which they consistently suffer from. Firstly, they just try to make the girth larger rather than decide the value of it, so the LDPC codes they constructed still suffer the loss of performance caused by short cycles. It would be unrealistic to raise the girth further beyond their capacity because of unbearable computational costs. Secondly, the quality of LDPC codes constructed via them has an over-reliance on their inputs, whereas excellent inputs are rarely available.

To solve these problems, we put forward an algorithm for constructing LDPC codes with arbitrary girth, and we call the algorithm the girth-cycle-embedding (GCE) algorithm. The algorithm requires the girth as the only input, and the code is constructed by embedding girth-member cycles into it. Our algorithm can overcome the above disadvantages through theoretical analysis, and produces LDPC codes with better performance than codes from PEG-based and QC-based algorithms in practice.

The rest of the paper is organized as follows: In Section 2, we introduce the representations of LDPC code, the concept of the cycle and its impact on LDPC code. In Section 3, PEG-based and QC-based algorithms are reviewed in detail. Section 4 depicts the GCE algorithm we proposed. Section 5 gives the performance evaluation of GCE, PEG-based and QC-based algorithms. The conclusion are presented in Section 6.

## 2. Preliminaries

In LDPC codes, a cycle is a path which alternately passes through check nodes and variable nodes [18] and ends at the same node it starts from. As an important factor, the cycle significantly influences the performance of an LDPC code. There is a large volume of published studies [23,24,25] indicating that shorter cycles are more harmful to the codes. When decoding, the circulation of information among different nodes is beneficial to error correction. However, the circulation of information is hindered by the existence of cycles, thus wrong information in cycles can not be updated by extrinsic information in time and makes errors difficult to correct. As the length of cycles gets shorter, the frequency of wrong information being recycled gets higher and the difficulty of error correction becomes greater. The length of the shortest cycles in an LDPC code is called the girth. An LDPC code with a large girth means that there are no cycles with lengths smaller than the girth, so numerous LDPC code construction algorithms have a very important goal, maximizing the girth. The hazard level of a cycle should be measured not only by its length but also by its connectivity [23,26,27] which measures the impact of extrinsic paths on the cycle. Currently, extrinsic message degree (EMD) [23], approximate cycle EMD (ACE) [26], etc., are used to quantify the connectivity. For two cycles of the same length, the cycle with higher connectivity receives more extrinsic information via the extrinsic paths, thereby breaking the information barrier faster. In other words, cycles with higher connectivity are relatively harmless.

In view of the harm of short cycles, many LDPC code construction algorithms are designed along the lines of maximizing the girth of LDPC codes, i.e., trying to avoid generating or to eliminate short cycles, and improving the connectivity of the short cycles when they cannot be avoided or removed. Next, we will introduce two main types of construction algorithms.

## 3. Construction Algorithms of LDPC Codes

There are many construction algorithms of LDPC codes, among which PEG-based and QC-based algorithms are the two main kinds of methods.

In the PEG-based algorithms, all the variable nodes are assigned targeted degrees by a variable-node degree distribution [18,28]. Then, for each variable node $v_{i}$, edges are greedily added into the LDPC code to maximize the length of the shortest cycle which $v_{i}$ participates in such that the algorithms manage to make the girth larger. The primary improvement direction of PEG-based algorithms is to develop better selection criteria, with which a variable node can decide which check nodes to connect with edges. For instance, in the PEG algorithm [18] the check node with the minimum degree will be chosen; in the improved PEG algorithm [19], it selects the check node with the highest cycle connectivity from check nodes with the minimum degree; generalized PEG algorithm [20] has harsher criteria: highest cycle connectivity, shortest paths, minimum degree, etc. These PEG-based algorithms are flexible and convenient to construct short-length and medium-length LDPC codes. In addition, both regular and irregular LDPC codes can be generated. Moreover, the codes created by these algorithms with an excellent variable-node degree distribution perform very well, especially in the waterfall region [29] which is signal noise ratio (SNR) or bit error rate (BER) region near the code threshold.

As for the QC-based algorithms, a matrix (LDPC code) is divided into many square matrices of the same size. Each of these square matrices is either a zero matrix or a circulant permutation matrix, which is obtained by cyclically right-shifting an identity matrix by *p* positions and *p* is called the shift of this circulant permutation matrix. Eliminating short cycles can be realized by giving suitable shift *p* for every circulant permutation matrix. In [21], a Hill-Climbing algorithm was proposed to greedily adjust those shift values to create a QC-LDPC code. In [22], the Hill-Climbing algorithm was improved in computational cost and the quality of matrices. These QC-based algorithms can remove cycles and are easy for hardware parallel implementation. The QC-LDPC codes they constructed save storage space and perform well in the error-floor region [30] which is the region with high SNR or with low BER.

Through the reviews of the PEG-based and QC-based algorithms above, we know that they remain the mainstream approaches to constructing LDPC codes because of many advantages. Despite these strengths, they consistently suffer from several shortcomings which are difficult to overcome.

Firstly, we found that all the PEG-based algorithms have an over-reliance on variable-node degree distribution [18,28]. It is one of the inputs of these algorithms and is used to decide the degree of each variable node. When too many variable nodes are assigned small degrees in one variable-node degree distribution, LDPC codes constructed may not provide enough information to help with error correction. However, increasing the number of variable nodes with large degrees is fairly easy to cause lots of short cycles. Therefore, variable-node degree distribution balancing degrees of all the variable nodes directly affects the quality of LDPC codes generated by the PEG-based algorithms. Several methods, e.g., density evolution [31,32] and Gaussian approximation [33], exist currently for creating variable-node degree distribution, but a major problem of them is that finite-length codes applying these methods only achieve suboptimal or general performance, and the performance even worsens for short-length codes. Thus, it is challenging to obtain an excellent variable-node degree distribution which is suitable for the code length you need. Secondly, there is no way to remove short cycles efficiently, because LDPC codes created by PEG-based algorithms are not structured and computational cost is prohibitive if all short cycles are detected and eliminated.

For structured LDPC codes generated by the QC-based algorithms, removing short cycles can be realized via changing the shifts. However, the computational cost needed grows exponentially with the increase of cycle length. Intuitively speaking, it is hard enough to discover and eliminate all eight-member cycles. Next, constructing a superb base matrix after determining its size is also a high-complexity question. Specifically, a m×n matrix (LDPC code) is divided into an M×N base matrix, and each element of the base matrix is a zero matrix or a Z×Z circulant permutation matrix, where Z=m/M=n/N. Therefore, there are ${\left(Z+1\right)}^{M\times N}$ possible combinations for the base matrix. At last, matrices from the QC-based algorithms suffer relatively poor performance compared with matrices from the PEG-based algorithms in the waterfall region.

Variants [34,35] of PEG-based and QC-based algorithms face similar drawbacks and have other disadvantages. For example, although the algorithm in [34] can construct LDPC codes with arbitrary girth, it reaches exponential complexity and can only generate regular LDPC codes [36,37].

## 4. Girth-Cycle-Embedding (GCE) Algorithm

As specified above, neither PEG-based nor QC-based algorithms can fully decide the girth so that the LDPC codes they constructed still suffer the loss of performance caused by short cycles. Furthermore, in these algorithms, the quality of LDPC codes is closely related to the inputs, i.e., variable-node degree distribution and base matrix, whereas excellent inputs are rarely available. In order to solve these problems, we put forward a new algorithm for LDPC code construction. In the algorithm, the expected girth is designed as the only input, and an LDPC code is constructed by way of embedding girth-member cycles into it. The algorithm overcomes the disadvantages mentioned above and produces LDPC codes with better performance than ones from PEG-based and QC-based algorithms. We call the algorithm we proposed GCE algorithm.

In the GCE algorithm, we denote the girth by *g* which can be expressed as g=2x because the length of any cycle must be an even number. To aid in managing all nodes, *m* check nodes are split into two sets, $cn_{new}$ for check nodes with zero degree while $cn_{old}$ for the others. In like manner, *n* variable nodes are divided into $vn_{new}$ and $vn_{old}$. In addition, we have designed an operation called FindTwoNode with dist as an input, where two nodes whose distance is dist are chosen and exported. In FindTwoNode operation, support tree [18,23] spreading from one check node as the root is used. For ensuring that a support tree has finite layers, all the nodes only appear in the tree once. Pseudocode of FindTwoNode operation is given in Algorithm 1.
**Algorithm 1**FindTwoNode.1:$C\left[0\cdots |cn_{old}|-1\right]⟵$ sort all the check node in $cn_{old}$ in ascending order of degree2:**for**j=0 to $|cn_{old}|-1$ **do**3:    $c_{j}⟵C\left[j\right]$4:    **if** the highest layer of $Tree(c_{j})\geq dist$ **then**5:        $\tilde{n}⟵$ randomly select one node with the minimum degree on $dist^{th}$ layer6:        **output:** $c_{j}$ and $\tilde{n}$7:    **end if**8:**end for**9:**output:** failure

In FindTwoNode operation, one of two nodes is a check node from $cn_{old}$, the other is also a check node from $cn_{old}$ if dist is even or a variable node from $vn_{old}$ if dist is odd. The detailed operation is as follows: sort all the check node in $cn_{old}$ based on the degree in ascending order; get a check node $c_{j}$ in order and build a support tree spreading from $c_{j}$ which is denoted by $Tree(c_{j})$; if $Tree(c_{j})$ can grow to the $dist^{th}$ layer, randomly select one node $\tilde{n}$ with the minimum degree on this layer, and output $c_{j}$ and $\tilde{n}$; if not, build the support tree of the next check node; the operation is considered a failure when all the support trees are less than dist layers.

After illustrating the FindTwoNode operation, we will introduce the GCE algorithm in detail. GCE is divided into four steps, each of which is a process of embedding girth-member cycles into the LDPC code in different ways as below. The pseudocode and an example of the GCE algorithm are shown in Algorithm 2 and Appendix A, respectively.

1. **Initialize node sets and form the first cycle.**
  Initialize the node sets: $cn_{new}=\left\{0\cdots m-1\right\}$, $vn_{new}=\left\{0\cdots n-1\right\}$, $cn_{old}=vn_{old}=\emptyset$. Then fetch x=g/2 check nodes from $cn_{new}$ and *x* variable nodes from $vn_{new}$, to form a girth-member cycle followed by putting these nodes into $cn_{old}$ and $vn_{old}$, respectively.
2. **Exhaust check nodes in** ${\mathrm{cn}}_{\mathrm{new}}.$
  Set a constant *h*:
  (1) $$h=\left\{\begin{array}{cc} x/2-1 & \left(x\;\mathrm{is}\;\mathrm{even}\right)\\ (x-1)/2 & \left(x\;\mathrm{is}\;\mathrm{odd}\right) \end{array}\right..$$
  If $|cn_{new}|$ is greater than or equal to *h*, execute FindTwoNode(x) when *x* is even or FindTwoNode(x−1) when *x* is odd. If FindTwoNode exports $c_{j}$ and $\tilde{n}$, connect $c_{j}$ and $\tilde{n}$ to create some girth-member cycles with *h* check nodes from $cn_{new}$ and h+1 variable nodes from $vn_{new}$, and put these *h* check nodes and h+1 variable nodes into $cn_{old}$ and $vn_{old}$, respectively. Then repeat step 2.
  If $|cn_{new}|$ is positive and less than *h*, execute $FindTwoNode(2(x-|cn_{new}\left|-1)\right)$. If FindTwoNode exports $c_{j}$ and $\tilde{n}$, connect $c_{j}$ and $\tilde{n}$ to create some girth-member cycles with $|cn_{new}|$ check nodes from $cn_{new}$ and $|cn_{new}|+1$ variable nodes from $vn_{new}$, and put these $|cn_{new}|$ check nodes and $|cn_{new}|+1$ variable nodes into $cn_{old}$ and $vn_{old}$, respectively. Then skip to step 3.
  If $|cn_{new}|$ is zero, then skip to step 3.
3. **Exhaust variable nodes in** ${\mathrm{vn}}_{\mathrm{new}}.$
  If $|vn_{new}|$ is a positive number, then execute FindTwoNode with input (2x−2). If FindTwoNode exports $c_{j}$ and $\tilde{n}$, connect $c_{j}$ and $\tilde{n}$ with a variable node from $vn_{new}$ to create some girth-member cycles, and put the variable node into $vn_{old}$. Then repeat step 3.
  If $|vn_{new}|$ is zero, then skip to step 4.
4. **Increase the degrees of variable nodes.**
  Execute FindTwoNode(2x−1). If FindTwoNode exports $c_{j}$ and $\tilde{n}$, connect $c_{j}$ and $\tilde{n}$ directly. Repeat step 4 until FindTwoNode fails.

**Algorithm 2** Girth-cycle-embedding algorithm.
1:Form the first *g*-member cycle2:**while** check nodes have not been exhausted **do**3:    **if** $|cn_{new}|\geq h$ **then**4:        **if** xmod2=0 **then**5:           $c_{j},\tilde{n}\leftarrow FindTwoNode\left(x\right)$6:        **else**7:           $c_{j},\tilde{n}\leftarrow FindTwoNode\left(x-1\right)$8:        **end if**9:    **else**10:        $c_{j},\tilde{n}\leftarrow FindTwoNode(2(x-|cn_{new}\left|-1)\right)$11:    **end if**12:    Connect $c_{j}$ and $\tilde{n}$ to form *g*-member cycles13:
**end while**
14:**while** variable nodes have not been exhausted **do**15:    $c_{j},\tilde{n}\leftarrow FindTwoNode\left(2x-2\right)$16:    Connect $c_{j}$ and $\tilde{n}$ to form *g*-member cycles17:
**end while**
18:**while**$c_{j},\tilde{n}\leftarrow FindTwoNode\left(2x-1\right)$ succeeds **do**19:    Connect $c_{j}$ and $\tilde{n}$ directly20:
**end while**



Except for step 1, steps 2–4 are executed with the help of FindTwoNode operation. Cycles with lengths less than *g* are avoided by setting the input of FindTwoNode reasonably, hence no more operations are needed to remove these cycles. As for cycles with lengths greater than or equivalent to *g*, their harm is significantly reduced by improving their connectivity in steps 2–4. Moreover, the only uncertain input in the GCE algorithm is the girth *g*, which greatly lowers the external influence on the quality of LDPC codes. The advantages above are shown directly in the experiments. Of course, GCE remains some shortcomings, for example leading to too small average variable-node degree and weakening the performance if *g* is too large or too small. However, compared with obtaining an excellent variable-node degree distribution for the PEG-based algorithms and a superb base matrix for the QC-based algorithms, the complexity of selecting an optimal *g* in GCE algorithm is low enough.

For an algorithm, it needs to consider space complexity and time complexity. Generally, the O-notation can be used to denote the asymptotic upper bound of space or time complexity [38]. We calculate the space and time complexity of GCE algorithm, some PEG-based and QC-based algorithms [18,19,20,21,22], and present the results in Table 1. It is observed that the GCE algorithm is not as good as the QC-based algorithms [21,22] in terms of space complexity, but it also remains linear. As for time complexity, the GCE algorithm has the lowest one compared to other algorithms. This indicates that the GCE algorithm has the least computational consumption when constructing an LDPC code in the asymptotic case.

## 5. Simulation Results

In this section, we executed three experiments to verify the advantages of the GCE algorithm. Before the experiments, we have constructed six matrices (LDPC codes) with code rate 1/2 (3072 check nodes and 6144 variable nodes) for the experiments. Three of them were created by using three PEG-based algorithms, i.e., PEG algorithm [18], improved PEG algorithm [19] and generalized PEG algorithm [20]. The variable-node degree distribution required was obtained by density evolution [31,32] and published in [32]. In QC-based algorithms, the Hill-Climbing algorithm [21] and the improved Hill-Climbing algorithm [22] were utilized to construct two matrices which both had 3×6 base matrices initialized in a random manner. The last matrix was generated via the GCE algorithm we proposed in Section 4 and its girth *g* was set to 12.

### 5.1. Decoding Performance

**Figure 1:**
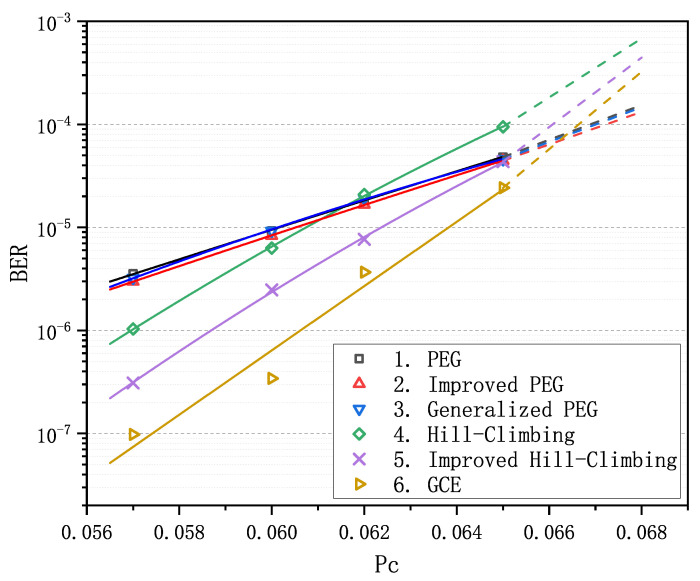
Comparison of decoding performance of the progressive-edge-growth (PEG)-based codes, quasi-cyclic (QC)-based codes and the code from the girth-cycle-embedding (GCE) algorithm at different $P_{c}$ values over binary symmetric channel (BSC).

Decoding with LDPC codes can correct errors in messages and thus guarantee the communication reliability of WSNs. For the comparison of decoding performance of finite-length LDPC codes, we chose to perform decoding simulations instead of running some analysis algorithms [39,40] which are inapplicable to the GCE algorithm. We first evaluated the decoding performance of the above six finite-length matrices with BP decoder introduced in [16,17] on the binary symmetric channel (BSC). Figure 1 presents the results with BER as the function of crossover probability $P_{c}$. For the convenience of reporting the results, all the matrices are numbered consecutively from 1 to 6: 1. PEG algorithm [18]; 2. improved PEG algorithm [19]; 3. generalized PEG algorithm [20]; 4. Hill-Climbing algorithm [21]; 5. improved Hill-Climbing algorithm [22]; and 6. GCE algorithm. It is apparent that there is not much difference in the performances of PEG-based matrices 1–3, while for two QC-based matrices, matrix 5 performs better than matrix 4.

**Figure 2:**
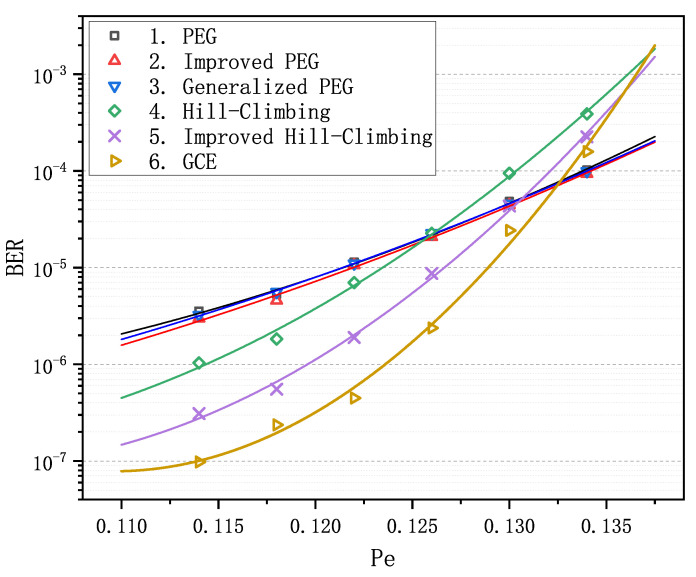
Comparison of decoding performance of the PEG-based codes, QC-based codes and the code from the GCE algorithm at different erasure probability $P_{e}$ over binary erasure channel (BEC).

**Figure 3:**
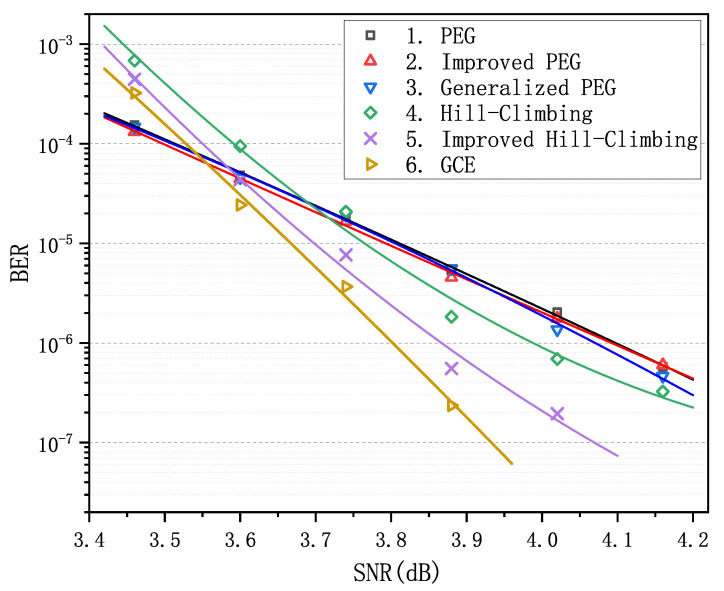
Comparison of decoding performance of the PEG-based codes, QC-based codes and the code from the GCE algorithm at different SNR over additive white Gaussian noise channel (AWGNC).

We observe that matrix 6 achieves the optimal performance when $P_{c}$ is less than 0.066. Particularly in the error-floor region, the gaps between the curve of matrix 6 and the others are rather striking. For example, when $P_{c}$ equals to 0.057, BER is $\left(3.26\pm 0.28\right)\times 10^{-6}$ for matrices 1–3 and is $9.77\times 10^{-8}$ for matrix 6, which span more than an order of magnitude. Obviously, as $P_{c}$ increases and goes into the waterfall region, matrix 6 gradually loses its advantage in decoding performance compared with matrices 1–3, nevertheless it still remains absolutely dominant compared with matrices 4 and 5. In order to verify the LDPC codes constructed by GCE outperform the LDPC codes constructed by other algorithms on different channels, we carried out two more decoding experiments over the binary erasure channel (BEC) and the additive white Gaussian noise channel (AWGNC), which are presented in Figure 2 and Figure 3, respectively. It is apparent that GCE algorithm surpasses the other algorithms in the error-floor region over different channels, which suggests that LDPC codes from GCE algorithm can provide more reliable communication for WSNs. The reason for outstanding decoding performance of the GCE algorithm is analyzed in Appendix B.

### 5.2. Power Consumption

**Figure 4:**
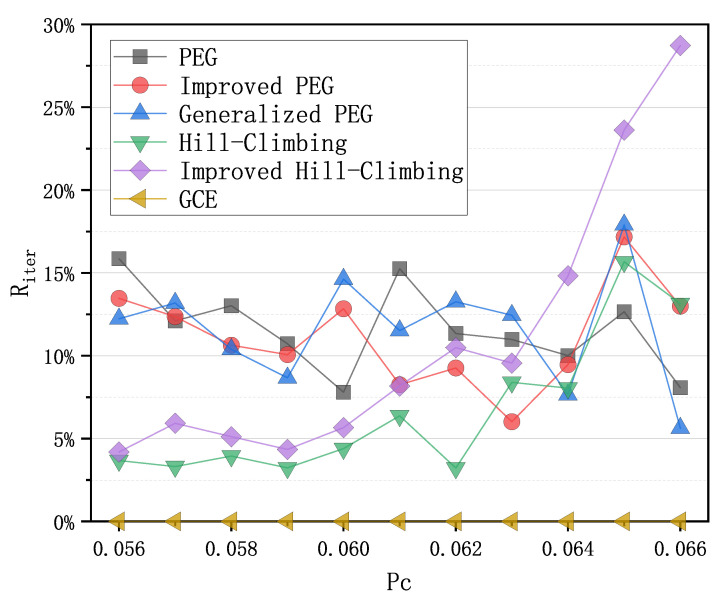
Comparison of iteration numbers of the PEG-based codes, QC-based codes and the code from the GCE algorithm with $R_{iter}$ from Equation (2) as the function of $P_{c}$ over binary symmetric channel (BSC).

Since the power of sensor nodes is limited, LDPC codes that achieve the same decoding effect but consume less energy are needed. Power consumption for decoding can be measured by the iteration number of BP decoder. A good LDPC code can effectively reduce the iteration number of BP decoder and such save energy. Therefore, we performed an experiment to calculate the average iteration numbers corresponding to the PEG-based codes, QC-based codes and the code from GCE algorithm, and present the results in Figure 4 with Equation (2) below,

(2) $$R_{iter}=\left(I_{algorithm}-I_{GCE}\right)/I_{GCE},$$

where $I_{algorithm}$ is the average iteration number with an LDPC code from one construction algorithm of LDPC codes, and $I_{GCE}$ is the average iteration number with an LDPC code from the GCE algorithm. From Figure 4, it can be seen that the LDPC code from GCE has the lowest iteration numbers at different crossover probabilities $P_{c}$. Assuming that the energy consumed per iteration is equal, the LDPC code from GCE can save 4% to 28% of energy compared to the LDPC codes obtained by other algorithms, which can effectively extend the lifetime of sensor nodes in WSNs.

### 5.3. Optimal Girth

**Figure 5:**
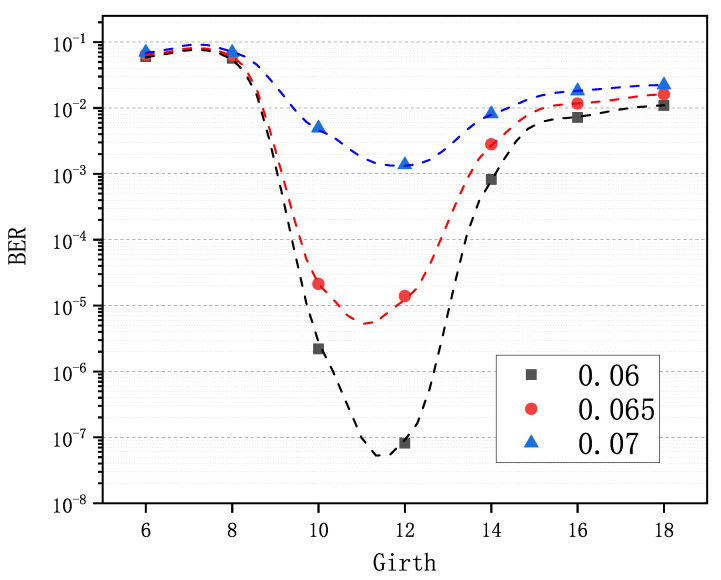
Comparison of decoding performance of the LDPC codes from the GCE algorithm with different girths (6–18) at different $P_{c}$ values (0.06, 0.065, 0.07).

An LDPC code construction algorithm is easy to wield, meaning that the user can easily get the optimal input to the algorithm. In Section 4, we know that compared with obtaining an excellent variable-node degree distribution for the PEG-based algorithms and a superb base matrix for the QC-based algorithms, it is much easier to find the optimal girth for the GCE algorithm, which is illustrated in Figure 5. In the test, seven matrices were generated via the GCE algorithm with girths *g* 6∼18, and $2\times 10^{3}$ key pairs were simulated for each of three $P_{c}$ values. Then, BER was calculated for each matrix and each $P_{c}$. As we can see in Figure 5, the optimal girths are the same, i.e., 12 for all of the $P_{c}$ values. The curves on both sides of the optimal girth are all monotonic. Therefore, by choosing one $P_{c}$ optionally and taking advantage of the monotonicity, we can approach and finally find the optimal girth for any code rate and any code length.

## 6. Conclusions

LDPC code is a good candidate for channel coding of WSN and can be constructed by PEG-based and QC-based algorithms. The aim of the present research was to discuss the ways to overcome the shortcomings of PEG-based and QC-based algorithms. We solve the problems by proposing GCE algorithm for constructing LDPC codes of any desired girth to avoid generating short cycles. The experimental result shows that the LDPC codes we construct have better decoding performance than the PEG-based and QC-based codes, especially in the error-floor region. LDPC codes constructed by GCE can effectively reduce the iteration number of decoding, thus reducing the power consumption of WSNs. In addition, the optimal input to GCE algorithm is easy to find.

## Figures and Tables

**Table 1:** Space and time complexity of different algorithms.

Algorithm	Space Complexity	Time Complexity
Progressive-edge-growth (PEG) [18]	O(m+n)	$O(n^{2})$
Improved PEG [19]	O(m+n)	$O(n^{2})$
Generalized PEG [20]	O(m+n)	$O(n^{2})$
Hill-Climbing [21]	O((m+n)/p)	O(mn/p)
Improved Hill-Climbing [22]	O((m+n)/p)	O(mn/p)
Girth-cycle-embedding (GCE)	O(m+n)	O(m+n)

**Figure A1:**
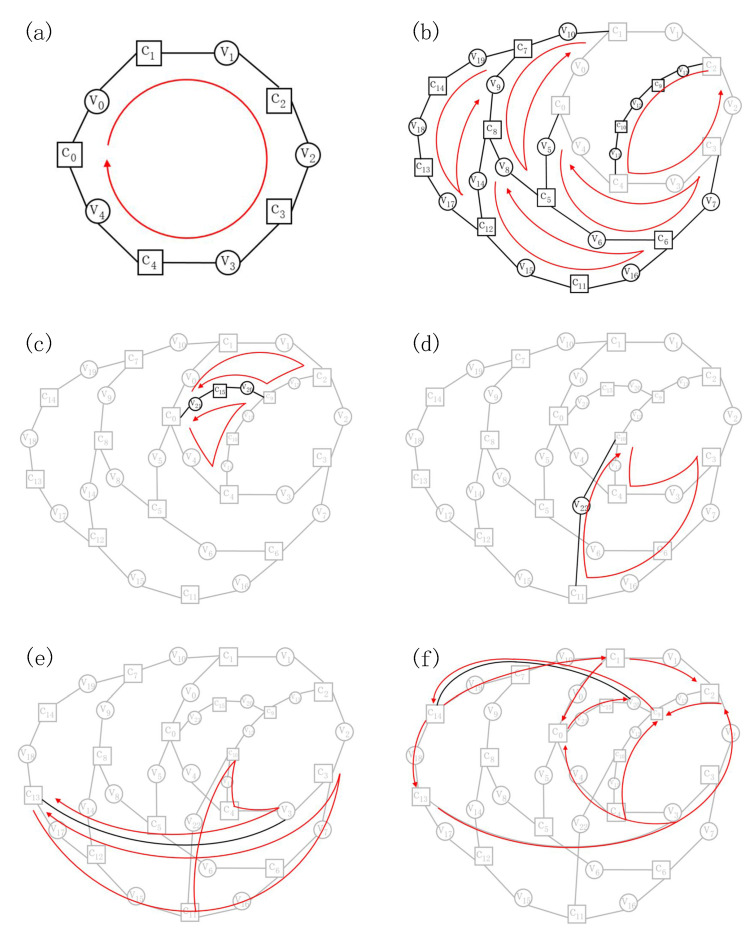
An example of constructing a 16×23 low-density parity-check (LDPC) code with girth 10 by GCE algorithm. (**a**) Initialize node sets and form the first cycle. (**b**,**c**) Exhaust check nodes in $cn_{new}$. (**d**) Exhaust variable nodes in $vn_{new}$. (**e**,**f**) Increase the degrees of variable nodes.

**Figure A2:**
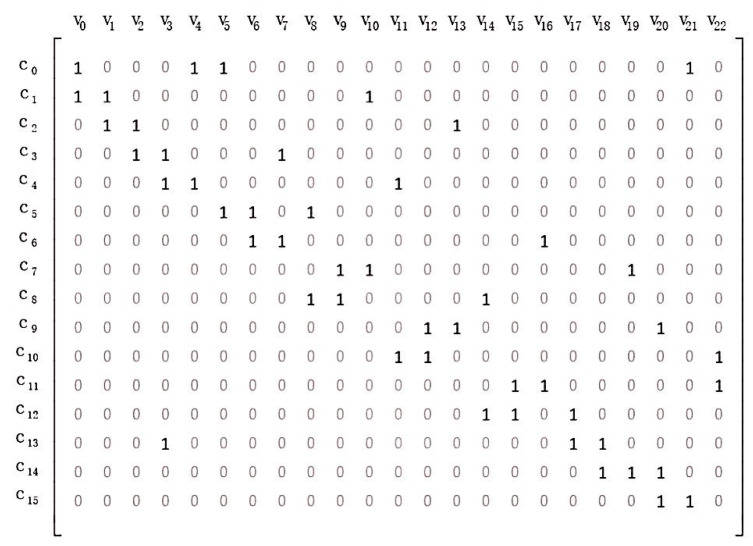
A 16×23 matrix transformed from the Tanner graph in Figure A1f.

## Data Availability

The data presented in this study are available on request from the corresponding author. The data are not publicly available due to subjects’ personal privacy and patents. The model of the subjects was public.

## Abbreviations

The following abbreviations are used in this manuscript:

WSN	Wireless sensor network
PEG	Progressive-edge-growth
QC	Quasi-cyclic
LDPC	Low-density parity-check
BP	Belief-propagation
GCE	Girth-cycle-embedding
EMD	Extrinsic message degree
ACE	Approximate cycle extrinsic message degree
SNR	Signal noise ratio
BER	Bit error rate
BSC	Binary symmetric channel
BEC	Binary erasure channel
AWGNC	Additive white Gaussian noise channel

## Appendix A

In this section, we provide an example of constructing an LDPC code with 16 check nodes and 23 variable nodes by GCE algorithm. The input of the GCE algorithm, i.e., the girth of the code, is g=2x=10. For the convenience of presenting cycles, the LDPC code is represented by a non-standard Tanner graph [41] without restriction on the locations of nodes. In addition, all the nodes are numbered in the order in which they are used. The entire process of the example is shown in Figure A1. For each subgraph, black lines indicate the new part; gray lines represent the previous part; ten-member cycles containing the new part are displayed with red lines.

In the first step of GCE algorithm, we initialize the node sets $cn_{new}=\left\{0\cdots 15\right\}$, $vn_{new}=\left\{0\cdots 22\right\}$, $cn_{old}=vn_{old}=\emptyset$, and form the first ten-member cycle as shown in Figure A1a with 5 check nodes and 5 variable nodes from $cn_{new}$ and $vn_{new}$, respectively.

In the second step, we execute FindTwoNode to find two check nodes, $c_{0}$ and $c_{3}$ whose distance is x−1=4, and fetch 2 check nodes from $cn_{new}$ and 3 variable nodes from $vn_{new}$ to connect $c_{0}$ and $c_{3}$, forming another ten-member cycle. Repeat the same action to find and connect four other check-node pairs: $\left(c_{1},c_{5}\right)$, $\left(c_{2},c_{4}\right)$, $\left(c_{6},c_{8}\right)$, $\left(c_{7},c_{12}\right)$, and obtain four other ten-member cycles as indicated in Figure A1b. So far, $|cn_{new}|=1<h=2$, and in order to exhaust the last check node we find $c_{0}$ and $c_{9}$ with distance 6 by FindTwoNode and connect them with the last check node and 2 variable nodes from $vn_{new}$. As presented in Figure A1c, two ten-member cycles are newly generated. Up to this point, all the check nodes have participated in the construction of the LDPC code, i.e., $|cn_{new}|=0$.

The result of the third step is plotted in Figure A1d. Two check nodes, $c_{10}$ and $c_{11}$ with distance 8, are exported by FindTwoNode and connected with the last variable node $v_{22}$ from $vn_{new}$. Thus all the variable nodes have been exhausted, i.e., $|vn_{new}|=0$.

In the forth step, we generate two ten-member cycles as shown in Figure A1e by connecting $c_{13}$ and $v_{3}$ with distance 9 directly. In like manner, we connect $c_{14}$ and $v_{20}$ in Figure A1f. So far, the distance between any check and variable nodes is less than 9.

A 16×23 LDPC code with girth 10 has been constructed after the steps above. We transform the Tanner graph in Figure A1f into a matrix provided in Figure A2.

## Appendix B

To find the reason for the outstanding decoding performance of GCE algorithm, for all the matrices in Section 5, we present their ACE spectra [26] associated with cycles of lengths 4–10 in Table A1 and their maximum degrees of variable nodes in Table A2. The ACE spectrum [26] of a matrix can be regarded as a vector where each element represents the minimum ACE of all the cycles with a certain cycle length. The minimum ACE of *l*-member cycles is positively related to the overall connectively of all the *l*-member cycles in the matrix. When there are no *l*-member cycles, the corresponding minimum ACE in the ACE spectrum is taken to be ∞.

**Table A1:** Approximate cycle extrinsic message degree (ACE) spectra of all the matrices.

	Length	4	6	8	10
Matrix					
**1**		∞	13	13	5
**2**		∞	27	13	10
**3**		∞	39	13	11
**4**		∞	∞	4	5
**5**		∞	∞	4	5
**6**		∞	∞	∞	∞

**Table A2:** Maximum degrees of variable nodes for all the matrices.

**Matrix**	1	2	3	4	5	6
**Degree**	15	15	15	3	3	7

As can be seen from Table A1, matrices 4 and 5 eliminate all six-member cycles while matrices 1–3 do not, which explains why matrices 4 and 5 outweigh matrices 1–3 when $P_{c}$ is less than 0.061. Moreover, it is noted that matrix 6 evades all four-member to ten-member cycles which can degrade the performance of any matrix, and thus outperforms the others. With the increase of $P_{c}$, however, the matrices with high-degree variable nodes gradually have the advantages. Connectivity of short cycles can be improved by raising the degrees of variable nodes to increase extrinsic paths (see Table A1), such that the hazard of short cycles is weakened. The variable nodes with high degrees can provide more decoding information to correct errors. In this respect, it is theoretically explained that matrices 1–3 with maximum degree 15 gradually perform better than matrices 4 and 5 with maximum degree 3 as displayed in Table A2. In addition, though matrix 6 has no cycles of lengths 4–10, its maximum degree is relatively smaller than matrices 1–3. Therefore, when $P_{c}$ gets into the waterfall region, matrix 6 works worse than matrices 1–3. In summary, the analysis above suggests that the matrices from the GCE algorithm we proposed have better decoding performance than the matrices constructed by PEG-based algorithms and QC-based algorithms, especially in the error-floor region.
